# Expression of Selenoprotein Genes and Association with Selenium Status in Colorectal Adenoma and Colorectal Cancer

**DOI:** 10.3390/nu10111812

**Published:** 2018-11-21

**Authors:** David J. Hughes, Tereza Kunická, Lutz Schomburg, Václav Liška, Niall Swan, Pavel Souček

**Affiliations:** 1 Cancer Biology and Therapeutics Group, UCD Conway Institute, University College Dublin, D04 V1W8 Dublin, Ireland; 2 Biomedical Centre, Medical and Teaching School Pilsen, Charles University in Prague, 323 00 Pilsen, Czech Republic; terez.kunicka@gmail.com (T.K.); vena.liska@skaut.cz (V.L.); pavel.soucek@szu.cz (P.S.); 3 Institute for Experimental Endocrinology, University Medical School Berlin, D-13353 Berlin, Germany; lutz.schomburg@charite.de; 4 Teaching Hospital and Medical School, Charles University in Prague, 306 05 Pilsen, Czech Republic; 5 Department of Pathology and Laboratory Medicine, St. Vincent’s University Hospital, D04 T6F4 Dublin, Ireland; n.swan@svuh.ie

**Keywords:** selenium (Se), selenoproteins, gene expression, selenium status, selenoprotein P, colorectal neoplasm, colorectal cancer, colorectal adenoma, biomarkers, cancer risk

## Abstract

Dietary selenium (Se) intake is essential for synthesizing selenoproteins that are important in countering oxidative and inflammatory processes linked to colorectal carcinogenesis. However, there is limited knowledge on the selenoprotein expression in colorectal adenoma (CRA) and colorectal cancer (CRC) patients, or the interaction with Se status levels. We studied the expression of seventeen Se pathway genes (including fifteen of the twenty-five human selenoproteins) in RNA extracted from disease-normal colorectal tissue pairs, in the discovery phase of sixty-two CRA/CRC patients from Ireland and a validation cohort of a hundred and five CRC patients from the Czech Republic. Differences in transcript levels between the disease and paired control mucosa were assessed by the Mann-Whitney U-test. *GPX2* and *TXNRD3* showed a higher expression and *GPX3*, *SELENOP*, *SELENOS*, and *SEPHS2* exhibited a lower expression in the disease tissue from adenomas and both cancer groups (*p*-values from 0.023 to <0.001). In the Czech cohort, up-regulation of *GPX1*, *SELENOH*, and *SOD2* and down-regulation of *SELENBP1*, *SELENON*, and *SELENOK* (*p*-values 0.036 to <0.001) was also observed. We further examined the correlation of gene expression with serum Se status (assessed by Se and selenoprotein P, SELENOP) in the Irish patients. While there were no significant correlations with both Se status markers, *SELENOF*, *SELENOK*, and *TXNRD1* tumor tissue expression positively correlated with Se, while *TXNRD2* and *TXNRD3* negatively correlated with *SELENOP*. In an analysis restricted to the larger Czech CRC patient cohort, Cox regression showed no major association of transcript levels with patient survival, except for an association of higher *SELENOF* gene expression with both a lower disease-free and overall survival. Several selenoproteins were differentially expressed in the disease tissue compared to the normal tissue of both CRA and CRC patients. Altered selenoprotein expression may serve as a marker of functional Se status and colorectal adenoma to cancer progression.

## 1. Introduction

Worldwide, colorectal cancer (CRC) is the second most common cancer in women and the third in men, with highest prevalence in developed countries [1]. Modifiable dietary and lifestyle patterns are important contributors to CRC etiology [2].

In humans, the essential micronutrient selenium (Se) is incorporated as the amino acid selenocysteine in twenty-five selenoproteins, of which several are involved in a wide variety of metabolic pathways implicated in carcinogenesis [3,4,5]. Observational and intervention studies suggest that Se status can influence colorectal adenoma (CRA) and colorectal cancer (CRC) development risk, particularly in the geographical areas of suboptimal Se availability, such as much of Europe [6,7]. Transcriptomic and proteomic studies demonstrate that Se intake can influence the pattern of selenoprotein expression and biosynthesis, affecting numerous oxidative stress, inflammatory, and signal translation pathways that are important in colorectal carcinogenesis [8,9,10]. Due to the hierarchical pattern of organ-specific selenoprotein expression in conditions of limited Se supply [3,11], tumor tissue specific expression patterns may provide a biologically informative marker of CRC risk, especially in relation to inadequate Se status [8,12].

However, there is limited data on the selenoprotein expression in CRA and CRC patient tissue, or the relationship with Se status. Human studies have generally only examined gene or protein colonic carcinoma tissue expression of key antioxidant selenoprotein P (SELENOP), glutathione peroxidase (GPX), and thioredoxin reductase (TXNRD) selenoenzymes that have different hierarchical expression regulation depending on dietary Se [3,8]. These studies have generally shown overlapping patterns of expression, with some differences in areas of lower [13,14] to higher [15] Se availability.

To examine the pattern of selenoprotein gene expression in CRA and CRC disease tissue and the surrounding non-neoplastic mucosa, the mRNA transcript expression level of seventeen Se pathway genes (including fifteen selenoproteins, the related *SELENBP1* Se biosynthesis gene, and the interacting *SOD2* antioxidant gene [16]) was evaluated for one hundred and sixty-seven Irish and Czech individuals (forty CRA and a hundred and twenty-seven CRC patients) by quantitative real-time PCR (qPCR). We were further able to compare the gene expression levels with two serum Se status markers (Se and selenoprotein P; SELENOP) in the Irish cohort. Potentially, selenoprotein mRNA level may be an easily measured molecular biomarker for assessing the biologically relevant Se status [17] for protection of healthy colonic tissue.

## 2. Materials and Methods

### 2.1. Patient Characteristics

This project consisted of a discovery and validation phase. In the discovery set of samples (Irish cohort), paired tumor tissue and adjacent non-neoplastic mucosa samples (and matched sera, where available) were obtained from a total of sixty-two patients with colorectal carcinoma (*n* = 22) or adenoma (*n* = 40), diagnosed at the Departments of Gastroenterology and Surgery, The Adelaide and Meath Hospital, in Dublin. The validation set (Czech cohort) comprised a hundred and five colorectal carcinoma patients, diagnosed at the Department of Surgery, Teaching Hospital and Medical School, in Pilsen, between January 2008 and November 2011. The clinical characteristics of our study cohorts are summarized in Table 1.

Irish tissue samples were collected during resection of primary tumor or by biopsy, before treatment, while adenoma biopsies were obtained at colonoscopy during a pilot CRC screening program as described previously [18]. Only advanced CRAs were included in the study—forty tubular or tubulovillous adenomas of at least 1cm, of which fourteen had high grade dysplasia (HGD), as the most screen-relevant lesions [19]. Blood samples were collected immediately within one day of surgery or colonoscopy, in plain 6 mL VACUTAINER^®^ tubes, (Cruinn Diagnostics, Dublin, Ireland) containing no anticoagulant. Blood was centrifuged at 2000× *g* for 10 min, within 4 h of collection, to separate the serum layer, which was then stored at −80 °C in cryovials. Collection and pathological processing of tissue samples and retrieval of data acquisition of the Czech samples, was performed as previously described [20]. Histology was verified by an experienced pathologist at each center. All CRCs were classified according to the tenth revision of the International Classification of Diseases (ICD-10) and the second revision of the International Classification of Disease for Oncology (ICDO-2). The clinical data, including age at diagnosis, sex, pTNM (Tumor stage, Regional lymph node involvement, and distant metastasis) staging, histological grade of the tumor, and primary tumor localization were taken from patient medical records (see Table 1).

All patients were asked to carefully read and sign an informed consent, in accord with the 1964 Helsinki Declaration. The study was approved by the Ethical Committee of the St. James’s Hospital and Federated Dublin Voluntary Hospitals Joint Research Ethics Committee (Ireland, reference 2007-37-17), and the Ethical Committee of the Medical Faculty and Teaching Hospital in Pilsen (Czech Republic, reference NT12025-4/2011). All samples were coded to protect patient anonymity.

### 2.2. Isolation of Total RNA

In the Irish cohort, the tissue samples were lysed on ice in 400 uL of lysis buffer (50 mmol/L HEPES, 4-(2-hydroxyethyl)-1-piperazineethanesulfonic acid, pH 7.5; 150 mmol/L NaCl; 5 mmol/L EDTA) and protease inhibitor (Calbiochem, Hampshire, UK) followed by sonication (3 × 30 s, on ice) and centrifugation (10,000× *g* for 10 min at 4 °C). Total RNA (as well as gDNA and protein) was then extracted using the Norgen All-in-one purification kit (Norgen, Thorold, ON, Canada). In the Czech cohort, tissue samples were homogenized by a mechanical disruption in liquid nitrogen using mortar and pestle, and the total RNA was isolated using the Trizol Reagent according to the manufacturer protocol (Invitrogen, Carlsbad, CA, USA). RNA was then stored at minus 80 °C and the quantity and quality were measured, as previously described [21].

### 2.3. Synthesis of Complementary DNA (cDNA)

Reverse transcription of the total RNA (0.5 µg for each reaction) was performed, using random hexamer primers and the RevertAid^TM^ First Strand cDNA Synthesis Kit (MBI Fermentas, Vilnius, Lithuania). Quality of the cDNA, in terms of DNA contamination, was confirmed by PCR amplification of *Ubiquitin C* [22].

### 2.4. Relative Quantification of Gene Expression

qPCR was performed using ViiA7 Real-Time PCR System, using TaqMan^®^ Universal Master Mix and TaqMan^®^ Gene Expression Assays (Life Technologies, Carlsbad, CA, USA) optimized primer and probe sets for the fifteen selenoprotein genes *SELENOP*, *SELENOS*, *GPX1-4*, *SELENOF*, *SEPHS2*, *SELENOH*, *SELENOK*, *SELENON*, *SELENOW*, *TXNRD1-3*, plus the Se biosynthesis gene *SELENBP1* and the interacting *SOD2* gene, in oxidative defense (Supplementary Table S1).

*POLR2A*, *PSMC4*, and *MRPL19* were used as the reference genes, based on stability assessment of twenty-four potential endogenous control genes in a test set of ten pairs of CRC tumors and non-neoplastic tissue samples [20]. Efficiency of the qPCR was determined for each assay using calibration curves (six-point, five times dilution), which were prepared from one non-neoplastic sample for the Czech cohort and from the mix of samples for the Irish cohort, respectively. The non-template control contained nuclease-free water instead of cDNA. Samples with variation larger than 0.5 Cq (quantitation cycle) were reanalyzed. The qPCR study adhered to the Minimum Information for Publication of Quantitative Real-Time PCR Experiments (MIQE) Guidelines [23].

### 2.5. Serum Selenium and Selenoprotein P Measurements

The sample type (cancer, adenoma, and control) was blinded. Concentrations of the total Se were measured in 4 µL of each serum sample, using a bench-top total reflection X-ray fluorescence (TXRF) spectrometer (PicofoxTM S2, Bruker Nano GmbH, Berlin, Germany), as described previously [7]. SELENOP levels were ascertained by a colorimetric enzyme-linked immunoassay (SelenotestTM, ICI GmbH, Berlin, Germany) using 5 µL of each serum sample in a 1:21 dilution, according to the manufacturer’s instructions [24]. Duplicate samples with differences in the Se concentration varying by more than 10% were measured again. The evaluation was performed with the GraphPad Prism 6.01 (GraphPad Software, La Jolla, CA, USA), using a four-parameter logistic function. The samples were measured in duplicate, and the mean concentration values, standard deviation (SD), and coefficients of variation (CVs) were calculated. The CVs were below 10% for the *SELENOP* controls 1 (1.5 mg/L) and 2 (8.6 mg/L) throughout the measurements.

### 2.6. Statistical Analyses

Transcript levels were analyzed by Viia7 System Software (Life Technologies, Carlsbad, CA, USA) and normalized levels of target genes (ratio of target gene Cq to the mean Cq of reference genes) were used for further statistical analyses, using SPSS v16.0 Software (SPSS Inc., Chicago, IL, USA). Differences in transcript levels between tumor and control tissues were assessed by nonparametric Mann-Whitney U-test. Nonparametric tests (the Kruskal-Wallis, the Mann-Whitney, and the Spearman’s test) were also used for evaluation of associations of transcript levels with clinical data and other variables (Table 1). Kaplan-Meier Log Rank test and multivariate Cox regression analyses, adjusted to stage, surgical radicality, and chemotherapy, were used to assess the gene transcript levels, with subject disease-free survival (DFS) and overall survival (OS), in the Czech CRC cohort. Cox regression was used to determine the hazard ratio (HR) and the 95% confidence intervals (95% CI) of the transcript levels, were divided according to the median, with these survival outcomes.

Analyses were conducted using SPSS v16.0 Software (SPSS Inc., Chicago, IL, USA). All *p*-values were obtained from two-sided tests; *p*-values lower than 0.05 were considered statistically significant. Multiple testing adjustment (*P_ADJ_*) was performed by the Benjamini-Hochberg correction (BH).

## 3. Results

### 3.1. Selenoprotein Gene Transcript Levels in the Colorectal Adenomas

In the Irish cohort, CRA patients showed a strongly significant lower expression of *SELENOP*, *SELENOS*, *GPX3*, and *SEPHS2* and higher expression levels of *GPX2*, *SELENOH*, and *TXNRD3* (*p* < 0.001 for all genes), in the disease tissue compared to the non-neoplastic control tissues. We tested the trend for expression with adenoma progression, compared to the normal tissues, by splitting the CRAs into two groups of twenty-six tubular and/or villous adenomas and another of fourteen HGDs. This suggested a declining expression of *SELENOP*, *SELENOS*, *GPX3*, and *SEPHS2* (respective *p*-values for trend at least 5.1 × 10^−5^) and increasing expression of *GPX2*, *SELENOH*, and *TXNRD3* (respective *p*-values at least 7 × 10^−5^), from the control to the increasingly dysplastic tissues.

### 3.2. Selenoprotein Gene Transcript Levels in Colorectal Cancers

In the tumor tissues of the CRC patients from Ireland, compared to the matched controls, *GPX2*, *GPX4*, *TXNRD3*, and *SOD2* were up-regulated (*p* = 0.023, 0.039, 0.003, and 0.036, respectively), while *SELENOP*, *SELENOS*, *GPX3*, *SEPHS2*, *SELENBP1*, and *SELENOK* were considerably down-regulated (*p* = 0.001, <0.001, <0.001, 0.002, <0.001, and 0.015, respectively).

In the validation study (Czech cohort), while many of the above observations were replicated, several disparities were observed. The Irish patients showed overexpressed levels of *GPX4* in tumors, compared to the control tissues, while the Czech subjects had no difference. Up-regulation of *GPX1* and *SELENOH* (*p* = 0.001 for both genes), and down-regulation of *SELENON* (*p* = 0.001) were demonstrated in the Czech patients, but no significant changes in the expression levels of these genes were found in the Irish cohort (although *SELENOH* was significantly more highly expressed in the adenoma tissue from the Irish CRA patients, *p* < 0.001).

All statistically significant correlations from the three groups of patients are summarized in Table 2. Directions of expression (higher or lower) were fully consistent for significant changes in the same gene across the different patient groups from Ireland and the Czech Republic. Gene expression levels did not correlate with the RNA integrity number (RIN), suggesting that RNA quality did not significantly influence the results.

**Figure 1:**
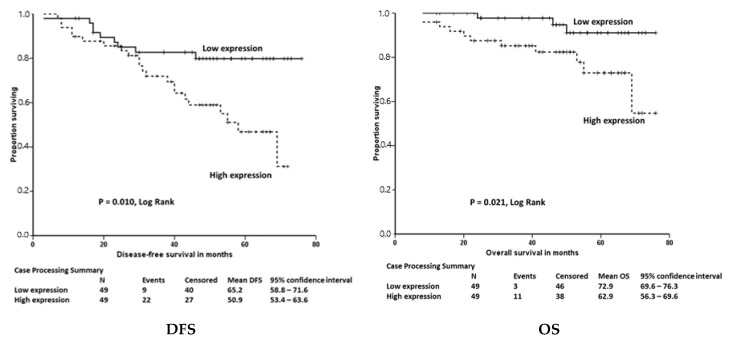
Association of the Selenoprotein F (*SELENOF*) gene expression with disease-free survival and overall survival, in colorectal cancer patients. Kaplan-Meier curves showing disease free survival (DFS) overall survival (OS) of 98 colorectal cancer (CRC) patients from the Czech Republic with higher *SELENOF* gene expression compared to low expression in tumor tissue. Subjects with higher *SELENOF* expression than median show poorer prognosis with shorter DFS and OS compared to subjects with lower *SELENOF* expression (50.9 vs. 65.2 months and 62.9 vs. 72.9 months, respectively; *P_DFS_* = 0.01 and *P_OS_* = 0.021). In multivariable Cox regression analyses adjusted by tumor stage, surgical radicality, and chemotherapy this was associated with a Hazards ratio (HR) point estimate of 2.41 for DFS (95% CI: 1.08, 5.41; *P* = 0.032) and 4.13 (95% CI: 1.10, 15.39; *P* = 0.035) for OS.

There were no major consistent differences in the expression patterns by age, sex, or tumor sub-site (colon/rectum) across the Irish and the Czech CRC samples. Stratified analyses—in the larger Czech CRC group—of expression by tumor stage, grade, and TNM status, showed no significant differences after the BH correction, except for an association of a higher cancer stage and presence of lymph node metastasis with decreased *SOD2* expression (*P_ADJ_* = 0.004 and *P_ADJ_* = 0.048, respectively). The presence of lymph node metastasis also significantly associated with decreased *GPX1* expression (*P_ADJ_* = 0.048). As our data indicated that increasing numbers of selenoprotein genes were dysregulated from the adenoma to tumor tissue and, thus, possibly to be involved in disease progression, we examined gene expression with cancer patient outcome. We performed analyses of tumor selenoprotein transcript levels, divided by the median, with the DFS and the OS, using 8–76 months patient follow-up data that were available for ninety-eight of the Czech CRCs (mean values of 58 and 68 months, respectively). Only a higher *SELENOF* expression was associated with both a poorer DFS and OS, in a Kaplan-Meier (see Figure 1) and multivariate-adjusted Cox regression analysis (HR: 2.41; 95% CI: 1.08, 5.41; *p* = 0.032 for DFS, and HR: 4.13; 95% CI: 1.10, 15.39; *p* = 0.035 for OS). However, this result, although biologically-plausible based on previous data of the oncogenic potential of *SELENOF* [3], should be cautiously considered, as the CIs were wide and the point estimates lost significance after multiple testing correction. Additionally, *SELENOF* did not show significant differences in overall expression levels between the neoplastic and the matched mucosal tissues.

Most of the studied genes (12 of the 17) showed non-significant expression changes in The Cancer Genome Atlas (TCGA) dataset (https://cancergenome.nih.gov/) (see Supplementary Figure S1). The other five genes showed concordant significant expression changes across the Irish, Czech, and the TCGA datasets, including the main Se transport protein-coding gene *SELENOP*, *GPX2*, *GPX3* (the second major Se transport gene), *TNRDX3,* and *SELENBP1* (see last column in Table 2). As presently we only had tissue RNA available for analysis, we were not able to analyze the protein expression in our samples. However, Supplementary Table S2 presents a summary of the immunohistochemistry protein expression data from the Human Protein Atlas (HPA) dataset (https://www.proteinatlas.org/), for the corresponding seventeen genes examined in this study. Although the numbers of the tumor samples with the available data are too small (*n* = 9–12 for each selenoprotein) to make meaningful inferences, several proteins (GPX2, GPX3, TXNRD3) had expression changes in most samples that matched the direction of gene expression variance observed in both the Irish and the Czech CRC samples. *SELENOP*, *SELENBP1*, and *SOD2* had medium protein expression levels, in both the normal and the tumor cells. Contrary results were given only by *SELENOS* and *SEPHS2*, which appeared to have relatively higher protein expression levels in the tumor cells (although *SEPHS2* was not detected in the normal cells).

### 3.3. Correlation of the Selenoprotein Transcript Levels with Se Status

Spearman correlation coefficients were calculated to ascertain the correlation of the selenoprotein gene expression in the Irish cohort with two measures of serum Se status (Se and SELENOP); available for thirteen CRA and seventeen CRC (Se) and thirty-eight CRA and eighteen CRC (SELENOP) patients, respectively. Only positive correlations of gene expression with the Se level were observed for the *SELENOF*, *SELENOK*, and *TXNRD1* genes, all in the tumor tissue (*p* = 0.001, 0.004, and 0.04, respectively), except for the normal tissue expression of *SELENOK* as well (*p* = 0.03). Regarding the SELENOP level, *TXNRD2* and *TXNRD3* tumor tissue expression was negatively correlated (*p* = 0.037 and 0.045), while *GPX1* had a higher expression in the normal tissue, *p* = 0.034. The only significant findings for adenomas were negative correlations for the SELENOP levels with *TXNRD1* disease (*p* = 0.006) and *SELENOW* normal (*p* = 0.042) tissue expressions. All correlation values are available in Supplementary Table S3.

## 4. Discussion

Expression profiles of seventeen Se pathway genes (including fifteen selenoproteins) were assessed in adenoma and cancer tissues, with their respective matched normal tissues, for forty CRA and a hundred and twenty-seven CRC patients. In comparisons between the neoplastic and normal tissue pairs, we observed seven differentially expressed genes in the CRA patients from Ireland and twelve dysregulated Se pathway genes for the CRC patients from the Czech Republic. Genes up-regulated in the tumor tissues were *GPX1*, *GPX2*, *SELENOH*, *TXNRD3*, and *SOD2*, while those down-regulated included *GPX3*, *SELENOP*, *SELENOS*, *SEPHS2*, *SELENBP1*, *SELENON*, and *SELENOK*. In adenomas *GPX2*, *SELENOH*, and *TXNRD3* also exhibited a higher expression in the disease tissue, while *GPX3*, *SELENOP*, *SELENOS*, and *SEPHS2* showed a lower expression for the Czech cancers. Thus, *GPX1*, *SELENBP1*, *SELENON*, *SELENOK*, and *SOD2* were differently expressed only in the cancer tissues.

Broadly similar expression patterns were observed for several selenoprotein genes (e.g., *GPX2*, *GPX3*, *SELENOP*, *SELENBP1*, and *TNRDX3*) across our Irish and Czech study groups, as compared with the gene and protein data for the TCGA and HPA, respectively. There were non-significant expression changes in the other selenoprotein genes in the TCGA, compared to significant findings in all our sample sets (*SEPHS2*, *SELENOS*), although the trend for a lower expression in tumor tissue for these genes was also seen in the TCGA. However, this is discordant with the corresponding SEPHS2 and SELENOS protein data in the HPA. These findings are probably largely explained by differences in the baseline Se status of the heterogenous populations in the TCGA and HPA data, resulting in differential regulation within the Se hierarchy [8], the method used for gene expression analysis (the more insensitive RefSeq, compared to the qPCR, for subtle changes), and variances in expression regulation at the gene or protein level.

Selenoproteins have several well-demonstrated or suggested cell protection roles in pathways implicated in colorectal carcinogenesis, such as the antioxidant response, immune, and inflammatory pathways [25,26,27], while downstream-targeted metabolic pathways are also affected by Se status, as demonstrated in human rectal biopsies [9]. Thus, altered selenoprotein expression in the colorectal tract may also be affected by Se status could increase cancer development risk by weakening the gut epithelial cell response to harmful oxidative and inflammatory challenges [8]. Transcriptomics animal studies highlight *Gpx1*, *Selenof*, *Selenoh*, and *Selenow* as being sensitive to Se supply and human Single Nucleotide Polymorphism (SNP) studies suggest *SELENOP*, *SELENOS*, *GPX4*, *SELENOF*, *SELENON*, *SELENOH*, and *TXNRD1-3* are key selenoproteins for colonic function and colorectal carcinogenesis [8,16,27,28]. However, prior to our investigation, data were lacking on the expression of all these selenoproteins in CRA and CRC. In this study, changes in gene expression were observed for *SELENOP*, *SELENOS*, and *TXNRD3* for all sample groups, while *GPX4* was significant in the Irish cancers only and *GPX1*, *SELENOH*, and *SELENON* were differently regulated in the Czech CRCs. Additional genes in this study, showing changes in gene expression for both the Irish and the Czech cancers were *GPX2*, *GPX3*, *SELENOK*, *SEPHS2*, *SELENBP1*, and *SOD2*.

Only a higher expression of the *SELENOF* gene was associated with patient outcomes from cancer (lower DFS and OS), although this was non-significant after multiple testing adjustment. However, several other lines of evidence suggest that *SELENOF* may act as an Se-dependent oncogenic protein. *SELENOF* can be regulated by both Se status (as indicated also in our study) and endoplasmic reticulum (ER) stress, and several *SELENOF* genetic variants have been associated with an altered risk at the different cancer sites, including CRC [8,29]. Notably, previous cell and mouse-model studies suggest that higher levels of *SELENOF* may potentially contribute to favorable growth conditions for the cancerous cells in the colorectum, by reducing cellular ER stress and nuclear factor kappa-light-chain-enhancer of activated B cells (NFkB) activation [29,30,31].

Normally, selenoprotein expression is sensitive to limited Se supply in a tissue-dependent manner [11]. As expected in conditions of redox stress, a lowered *SELENOP* expression was reported in studies of a small number of German CRA and CRC subjects, while heterogenous *GPX2* mRNA and protein expression was observed between the tumor samples [13,32]. In German patients, *GPX1*, *GPX4*, and *TXNRD1* were also found to be up-regulated in cancer, compared to the matched tissues for mRNA and/or their corresponding protein levels [14]. However, while *GPX1* and *GPX4* were more highly expressed in tumor tissue from the Czech and Irish cancer groups, respectively, *TXNRD1* was not significantly different in any of our tested cohorts. In other settings of generally higher Se availability, such as in Japan, the protein expression of GPX1, GPX3, and SELENOP was reported to be lower in the CRC tumors, whereas the less Se-sensitive GPX2 was increased [15]. We observed similar results for our mRNA assays for *GPX2*, *GPX3*, and *SELENOP* for all CRA and CRC groups.

Hypoxic and oxidative stresses in proliferating tumors may also decouple the normal hierarchy of selenoprotein expression [12]. *SELENOH* is a putative redox regulating DNA-binding protein, whose cellular expression is sensitive to Se supply [26,33]. In contrast, protein expression of SELENOH has recently been shown to be higher in CRC human tumors (and to control cell-cycle progression and tumor proliferation in mouse and human CRC cell-line models) [12], aligning with the upregulation of *SELENOH* in the CRAs and the Czech CRCs observed in this study. The Se binding protein 1 (*SELENBP1*) gene and corresponding protein expression was found to be downregulated in Chinese CRC samples, as observed for the *SELENBP1* mRNA levels in the cancer cohorts in this study [34].

Overall, the apparent expression pattern we assessed in the colorectal tumor tissue is that the genes related to Se homeostasis (*SELENOP*, *SEPHS2*, *SELENBP1*) and ER stress (*SELENOK*, *SELENOS*) are down-regulated, while the antioxidant enzymes might exhibit a higher (*GPX1*, *GPX2*, *SELENOH*, *TXNRD3*, *SOD2*) or lower (*GPX3*, *SELENON*) expression [3,6,8]. In a previous examination of the common genetic variation in selenoproteins and Se-pathway genes, we observed that several SNPs in many of these genes were associated with CRC risk [35]. These data all support a role of selenoprotein metabolism and endoplasmic and oxidative stress in CRC development. Differences and overlaps between studies likely reflect design issues of sample size and tissue stage and site sampled, or underlying biological differences in the Se metabolism. This could be due to population specific differential regulation within the Se hierarchy, depending on the Se availability and selenoprotein genetic variation [8].

Several selenoprotein genes showed sequential expression differences through the major stages of dysplastic adenoma progression observed in different individuals in the Irish study. These included increasing down-regulation of *GPX3*, *SELENOP*, *SELENOS*, and *SEPHS2* and up-regulation of *GPX2*, *SELENOH*, and *TXNRD3*, in groups of normal-matched tissues, adenomas, and adenomas with HGD. Additional and larger studies will help clarify the pattern of selenoprotein expression in the adenoma progression. Possibly, selenoprotein expression may have uses as markers of advanced adenoma stages, relevant for CRC screening, especially as only advanced adenomas appear to be associated with an increased risk for subsequent CRC development [36].

Suboptimal Se status levels are found in many parts of Europe [37,38]. Our measures of Se status were only possible for available matching serum samples for the Irish CRA and CRC patients. Here, the mean levels of Se and *SELENOP* (86.1 μg/L in 30 sera and 5.1 mg/L in 56 sera, respectively) were slightly higher, as compared to eight other Western European countries in a prospective study, showing an association of higher levels with a decreased CRC risk (85.6 μg/L and 4.4 mg/L, respectively for the controls [7]). Although, there were no marked correlation patterns between either of these serum Se status levels and the selenoprotein gene expression, they did indicate the potential importance of the Se supply for expression of *GPX1*, *SELENOF*, *SELENOK*, and *TXNRD1-3*. Most of the few significant correlations were observed for the tumor tissue gene expression, rather than adenoma tissue or the matched normal tissues for these pathologies, suggesting that Se status is a factor for the differential expression we observed for *SELENOK* and *TXNRD3* in the Irish CRC group. Although we did not observe significant correlations of their expression with the Se status, other selenoprotein genes whose activities are thought to be sensitive to lower Se status in the gastrointestinal tract, such as *SELENOP* and *SELENOH* [8,12], were also observed to be differentially regulated in the adenomas and/or cancers groups from Ireland. However, the total numbers available for these analyses were small and confined to the Irish samples. Therefore, this should be considered as an exploratory analysis requiring further investigation to more fully ascertain the degree to which any involvement of selenoprotein expression in affecting CRA to CRC development may depend on the Se status of the studied population.

In summary, several selenoprotein genes were differentially expressed in both the CRA and CRC disease-normal tissue pairs. These include key biological stress response genes like *GPXs*, *TXNRD3*, *SELENOS*, *SELENOH*, and the related *SOD2* gene implicated in cancer cell survival, and genes such as *SELENOP* and *SEPHS2* involved in Se biosynthesis. However, as only some of these expression changes were correlated with the Se status levels, selenoprotein expression may affect CRA to CRC development independent of the Se status. Functional studies will be required to further investigate any role of the identified differentially-expressed selenoproteins in colorectal carcinogenesis. Potentially, selenoprotein mRNA expression may have uses as biomarkers of colorectal function, colorectal neoplasia progression, and improved assessment of physiological Se status, with implications for the modulation of Se intake.

## Figures and Tables

**Table 1:** Clinical-pathological characteristics of the studied cohort of patients.

Cohort	Irish	Irish	Czech
Diagnosis	CRA	CRC	CRC
Total n of tissue samples	40	22	105
Sex n (male/female)	26/14	11/11	64/25
Age at diagnosis, median ± SD (years)	61 ± 7 years	59 ± 11 years	69 ± 11 years
Location n (colon/rectum)	28/12	15/7	82/17
T staging n (/T1/T2/T3/T4)	-	6/4/9/3	-
N staging n (N0/N1 or N2) (missing)	-	19/3	78/25 (2)
M staging n (M0/M1)	-	21/1	-
Stage (I/II/III/IV (missing))	-	10/7/4/1	2/76/15/10 (2)

SD = standard deviation; CRC = colorectal cancer; CRA = colorectal adenoma; - = not applicable or missing; TNM staging = Tumor stage, Regional lymph node involvement, and distant metastasis.

**Table 2:** Differences in the transcript levels of the examined genes between the colorectal adenoma/carcinoma and the non-neoplastic tissue in the Irish and the Czech CRA/CRC patients (this study), and comparison with data from the TCGA patient cohorts.

Gene Name	Discovery (Irish Cohort, *n* = 62; 40 CRA and 22 CRC)		Validation (Czech Cohort, *N* = 105 CRC) Carcinoma Expression (*p*-Value)	TCGA (COAD/READ) Carcinoma Expression
	Adenoma Expression (*p*-Value)	Carcinoma Expression (*p*-Value)		
*GPX1*	NS	NS	↑ (<0.001)	NS/NS
*GPX2*	↑ (<0.001)	↑ (0.023)	↑ (<0.001)	↑/↑
*GPX3*	↓ (<0.001)	↓ (<0.001)	↓ (<0.001)	↓/↓
*GPX4*	NS	↑ (0.039)	NS	NS/NS
*SELENOF*	NS	NS	NS	NS/NS
*SELENOH*	↑ (<0.001)	NS	↑ (<0.001)	NS/NS
*SELENOK*	NS	↓ (0.015)	↓ (<0.001)	NS/NS
*SELENON*	NS	NS	↓ (<0.001)	NS/NS
*SELENOP*	↓ (<0.001)	↓ (0.001)	↓ (<0.001)	↓/↓
SELENOS	↓ (<0.001)	↓ (<0.001)	↓ (<0.001)	NS/NS
*SELENOW*	NS	NS	NS	NS/NS
*SEPHS2*	↓ (<0.001)	↓ (0.002)	↓ (<0.001)	NS/NS
*TXNRD1*	NS	NS	NS	NS/NS
*TXNRD2*	NS	NS	NS	NS/NS
*TXNRD3*	↑ (<0.001)	↑ (0.003)	↑ (<0.001)	↑/↑
*SELENBP1*	NS	↓ (<0.001)	↓ (<0.001)	↑/↑
*SOD2*	NS	↑ (0.036)	↑ (<0.001)	NS/NS

Abbreviations: ↑ = higher expression in the neoplastic compared to the normal tissue, ↓ = lower expression in the neoplastic compared to the normal tissue, NS = not significant, TCGA = The Cancer Genome Atlas (https://cancergenome.nih.gov/), COAD = colon adenocarcinoma, and READ = rectum adenocarcinoma. For the seventeen genes tested in the Irish and the Czech studies, only *SELENOF*, *SELENOW*, *TXNRD1*, and *TXNRD2* were not significantly different in at least in one of the neoplasm groups. Genes significant in all three Irish and Czech sample sets (*GPX2*, *GPX3*, *SELENOP*, *SELENOS*, *SEPHS2*, and *TXNRD3*) are marked by a dark background. *p*-values are in brackets. TCGA data: Analysis done on the TCGA tumor-normal data, using the GEPIA (Gene Expression Profiling Interactive Analysis) tool (http://gepia.cancer-pku.cn/about.html; Tang et al. Nucleic Acids Res. 2017; 45(W1): W98–W102).

## Supplementary Materials

The following are available online at http://www.mdpi.com/2072-6643/10/11/1812/s1, Figure S1: Selenoprotein gene expression profiles from tumor-normal data of human colorectal tumor (red boxes) and mucosa (grey boxes) tissues from the TCGA database, Table S1: List of TaqMan selenoprotein and selenium-related gene expression assays used for this study, Table S2: Protein levels of selenoproteins in normal and colorectal cancer tissues in The Human Protein Atlas, Table S3: Spearman correlation values for tissue selenoprotein gene expression and serum selenium status in cancer and adenoma patients from Ireland.
