# Supplementary material for: Expression of Selenoprotein Genes and Association with Selenium Status in Colorectal Adenoma and Colorectal Cancer
Source: Nutrients. 2018 Nov 21;10(11):1812. doi: 10.3390/nu10111812 (PMC6266908; doi:10.3390/nu10111812)
Supplement: Supplementary file 1 [file nutrients-10-01812-s001.zip › Figure S1revised 240918.pptx]

## Slide 1
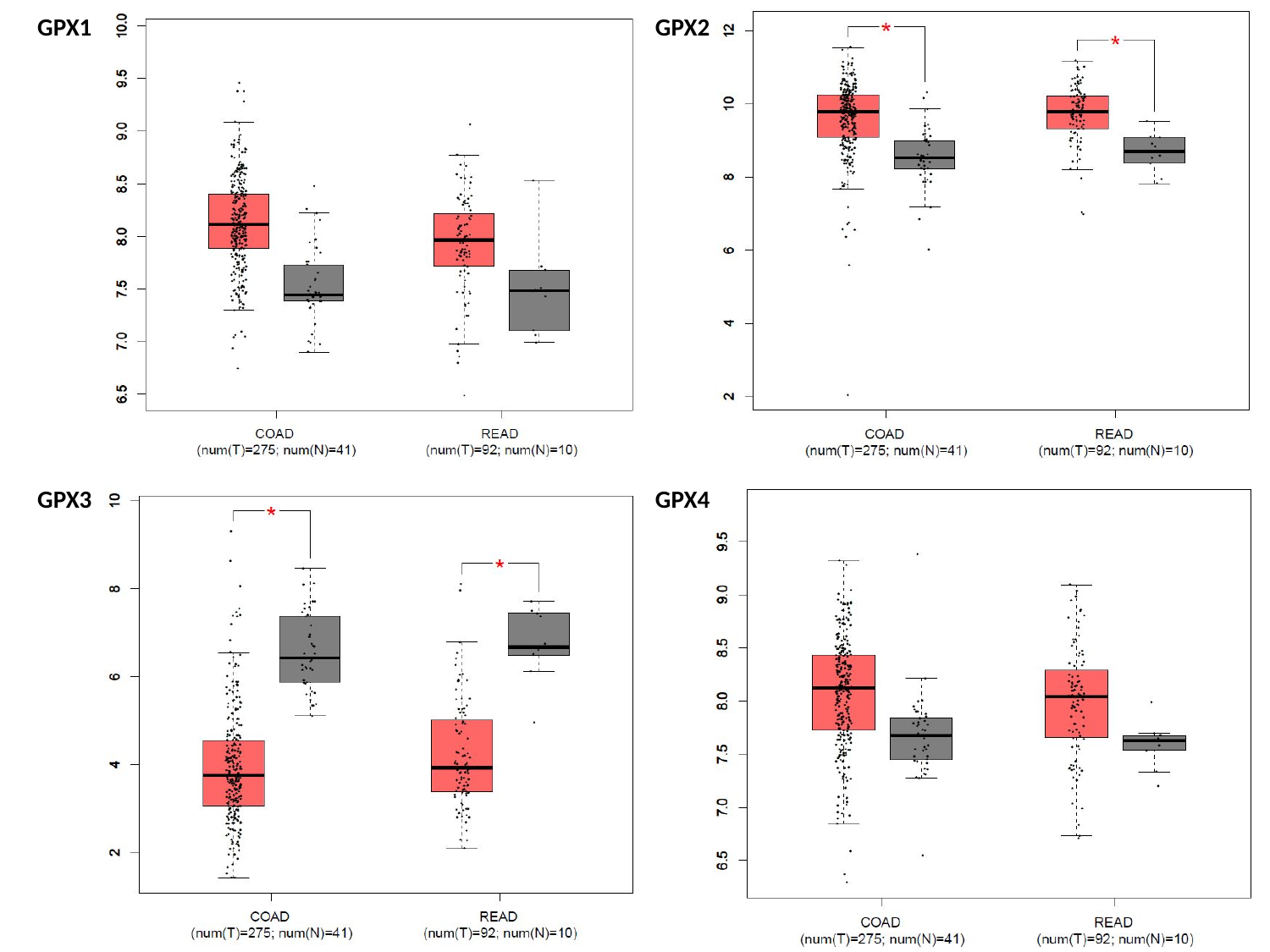

GPX1				 GPX2
GPX3				 GPX4

## Slide 2
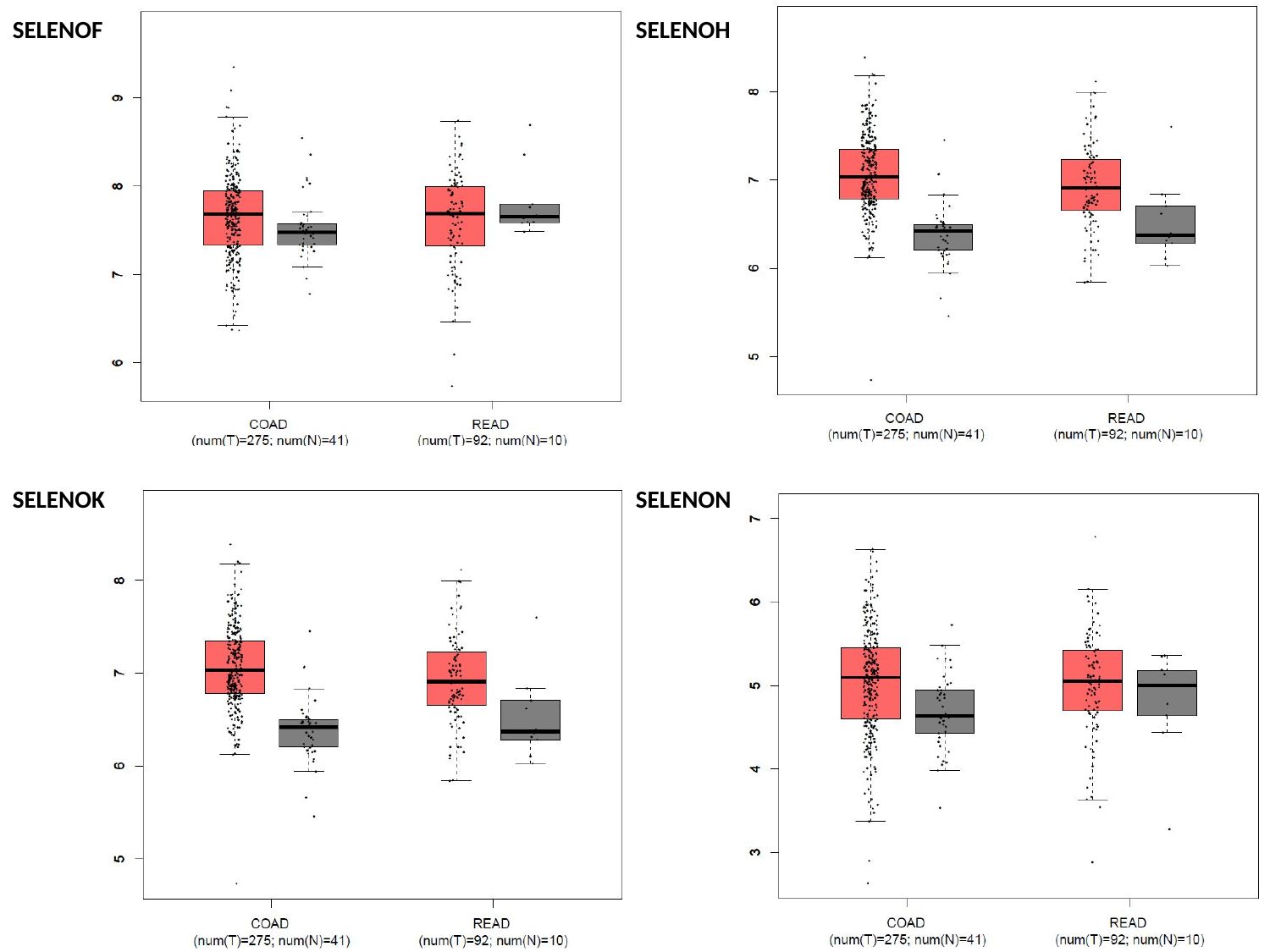

SELENOF				 SELENOH
SELENOK				 SELENON

## Slide 3
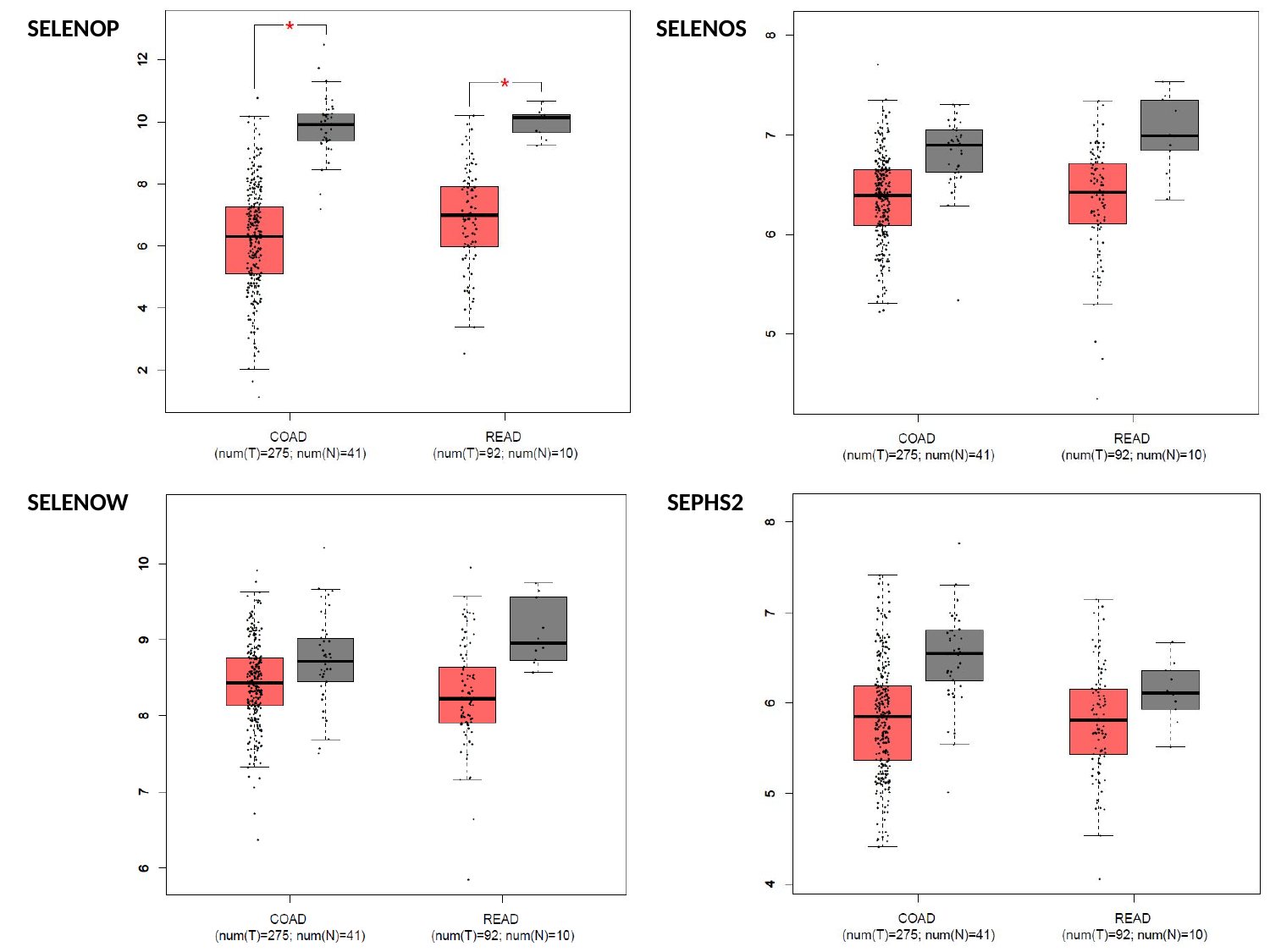

SELENOP				 SELENOS
SELENOW				 SEPHS2

## Slide 4
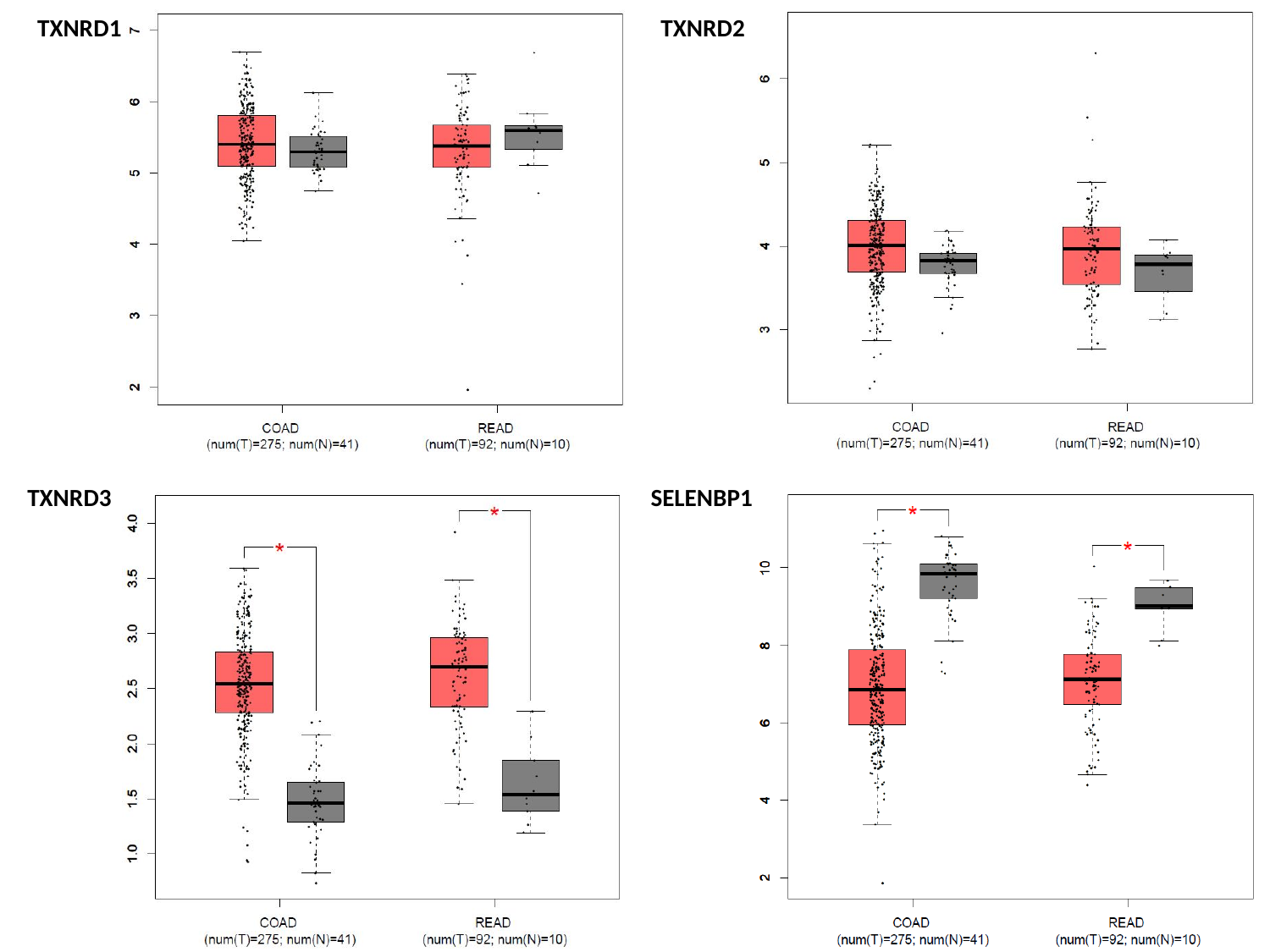

TXNRD1				 TXNRD2
TXNRD3				 SELENBP1

## Slide 5
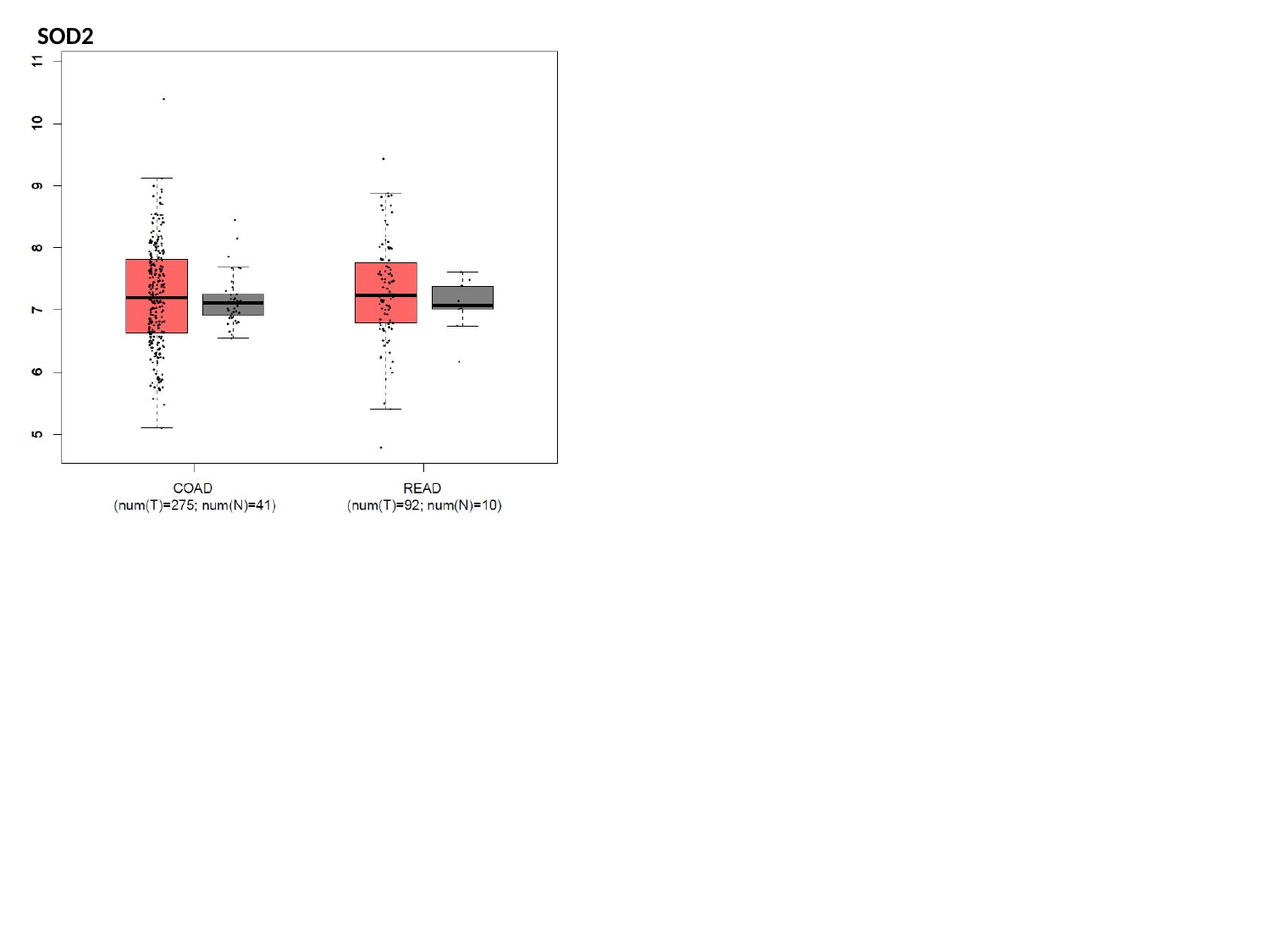

SOD2
