# Supplementary material for: Expression of Selenoprotein Genes and Association with Selenium Status in Colorectal Adenoma and Colorectal Cancer
Source: Nutrients. 2018 Nov 21;10(11):1812. doi: 10.3390/nu10111812 (PMC6266908; doi:10.3390/nu10111812)
Supplement: Supplementary file 1 [file nutrients-10-01812-s001.zip › Supplementary Figure S1 legend.docx]

**Supplementary Figure 1 legend:** Selenoprotein gene expression profiles from tumor-normal data of human colorectal tumor (red boxes) and mucosa (grey boxes) tissues from the TCGA database.

Results of gene expression profiling were visualized by GEPIA (Gene Expression Profiling Interactive Analysis) tool (http://gepia.cancer-pku.cn/, see Tang et al. Nucleic Acids Res. 2017;45(W1):W98-W102).

Footnotes: TCGA = The Cancer Genome Atlas (TCGA) dataset (https://cancergenome.nih.gov/), COAD = samples from colon adenocarcinomas, READ = samples from rectal adenocarcinomas; *P<0.05
