# Supplementary material for: Expression of Selenoprotein Genes and Association with Selenium Status in Colorectal Adenoma and Colorectal Cancer
Source: Nutrients. 2018 Nov 21;10(11):1812. doi: 10.3390/nu10111812 (PMC6266908; doi:10.3390/nu10111812)
Supplement: Supplementary file 1 [file nutrients-10-01812-s001.zip › Table S1 Selenoprotein gene assays.docx]

**Supplementary Table S1: List of *TaqMan* selenoprotein and selenium related gene expression assays used for this study.**

| **Assay ID** | Hs00172187_m1 | Hs00197826_m1 | Hs00608519_m1 | Hs01032845_m1 | Hs01104466_m1 | Hs00829989_gH | Hs01591589_m1 | Hs01078668_m1 | Hs00989765_g1 | Hs00907906_m1 | Hs01102833_s1 | Hs00187625_m1 | Hs00415057_m1 | Hs01071062_m1 | Hs00898723_m1 | Hs00431229_g1 | Hs00917067_m1 | Hs01561341_m1 | Hs01082598_m1 | Hs00167309_m1 |
| --- | --- | --- | --- | --- | --- | --- | --- | --- | --- | --- | --- | --- | --- | --- | --- | --- | --- | --- | --- | --- |
| **Gene name** | Polymerase (RNA) II (DNA directed) polypeptide A, 220kDa | Proteasome (prosome, macropain) 26S subunit, ATPase, 4 | Mitochondrial ribosomal protein L19 | Selenoprotein P, Plasma, 1 | Selenoprotein S (VCP-Interacting Membrane Protein) | Glutathione Peroxidase 1 | Glutathione Peroxidase 2 | Glutathione Peroxidase 3 | Glutathione Peroxidase 4 | 15 kDa Selenoprotein | Selenophosphate Synthetase 2 | Selenium Binding Protein 1 | Selenoprotein H (Chromosome 11 Open Reading Frame 31) | Selenoprotein W, 1 | Selenoprotein N, 1 | Selenoprotein K | Thioredoxin Reductase 1 | Thioredoxin Reductase 2 | Thioredoxin Reductase 3 | Superoxide Dismutase 2, Mitochondrial |
| **Gene symbol** | *POLR2A* | *PSMC4* | *MRPL19* | *SELENOP* | *SELENOS* | *GPX1* | *GPX2* | *GPX3* | *GPX4* | *SELENOF* | *SEPHS2* | *SELENBP1* | *SELENOH* | *SELENOW* | *SELENON* | *SELENOK* | *TXNRD1* | *TXNRD2* | *TXNRD3* | *SOD2* |
